# Utilization of psychotherapy, pharmacotherapy, and their combination by individuals with current or residual depression: results from five annual nationally representative German surveys

**DOI:** 10.1038/s41598-025-32371-6

**Published:** 2025-12-13

**Authors:** Louisa Breuer, Andreas Czaplicki, Ulrich Hegerl, Hanna Reich

**Affiliations:** 1 Depression Research Centre of the German Depression Foundation, Department of Psychiatry, Psychosomatic Medicine and Psychotherapy, University Hospital, Goethe University, Frankfurt am Main, Germany; 2 German Foundation for Depression and Suicide Prevention, Leipzig, Germany; 3 Department of Psychiatry, Psychosomatic Medicine and Psychotherapy, University Hospital Frankfurt, Goethe University Frankfurt (Goethe Research Professorship), Frankfurt am Main, Germany

**Keywords:** Depression, Psychotherapy, Pharmacotherapy, Representative survey, Self-report, Health services, Epidemiology

## Abstract

Depression is a common and burdensome mental disorder affecting more than 320 million people worldwide. Clinical practice guidelines recommend psychotherapy, antidepressant pharmacotherapy, or their combination as the cornerstones of evidence-based treatment. Despite these well-established therapeutic options, a substantial proportion of individuals with depression remain untreated. In Germany, available estimates of the current treatment gap are outdated. The present study therefore aims to examine report the utilization of psychotherapy, antidepressant pharmacotherapy, or their combination among adults in Germany who self-reported a diagnosis of depression. In addition, we explore potential differences in treatment utilization according to sociodemographic characteristics, specifically sex and age. Between 2018 and 2022, annual population-representative surveys were conducted in Germany. A total of *N* = 3,416 individuals who self-reported having been professionally diagnosed with depression at least once in their lifetime and who indicated current acute or residual symptoms were included in the present analysis. Overall, 63.4% of participants reported using at least one guideline-based treatment for their current depressive episode or residual symptoms. Specifically, 13.5% reported receiving psychotherapy, 26.5% pharmacotherapy, and 23.4% a combination of both. The likelihood of utilizing psychotherapy decreased with age (*18–29 years: OR = 1.22*,* 95% CI = 0.91–1.64; 60–69 years: OR = 0.36*,* 95% CI = 0.24–0.52)*, whereas the likelihood of pharmacotherapy use increased with age (*18–29 years: OR = 0*.*74*,* 95% CI = 0.54–1.01; 60–69 years: OR = 2*.*46*,* 95% CI = 1.89–3.23)*. Although self-reported utilization of guideline-based treatment for depression was higher than previously reported, depression in adulthood likely remains undertreated. These findings highlight the need to strengthen access to evidence-based interventions and to promote long-term treatment adherence and self-management strategies.

## Introduction

Affective disorders are highly prevalent worldwide, with unipolar depression being the most common subtype, affecting an estimated 16–20% of the global population^1,2^. In Germany, the 12-month prevalence of depression is approximately 7%^3,4^. Depressive disorders rank among the leading contributors to the global burden of disease^5,6^, and projections indicate that unipolar major depression may become the leading cause of disease burden worldwide by 2030^4,7,8^. Although depressive episodes may occur as single or recurrent events depending on clinical, personal, and social determinants^9^, a substantial proportion of patients fail to achieve full remission. Approximately one-third continue to experience residual symptoms despite adequate treatment, a condition often defined as treatment-resistant depression (TRD)^9^. These developments underscore a central challenge in contemporary mental healthcare: even in high-income countries with well-established treatment systems, persistent diagnostic, access, and adherence gaps sustain the societal and individual burden of depression^10^.

In Germany, a broad range of guideline-based therapeutic options for depressive disorders exist, including psychotherapy, antidepressant pharmacotherapy, or their combination as first-line interventions^1,11,12^. Additional modalities - such as electroconvulsive therapy (ECT), light therapy, or sleep deprivation therapy – may be used when initial treatments prove insufficient^13^. Yet despite this extensive therapeutic infrastructure, a substantial treatment gap remains. Across the WHO European Region, this gap has been estimated at 45.4% for major depression^14^, with comparable deficits reported in Germany^15,16^. This apparent discrepancy between treatment availability and treatment utilization represents a core theoretical tension in current health-services research: the existence of evidence-based interventions does not guarantee their uptake.

Both structural and individual factors contribute to this discrepancy. On the supply-side, long waiting times, insufficient recognition of depression, and limited structured pathways for TRD or difficult-to-treat depression can impede timely care^9^. On the demand-side, help-seeking behaviour is shaped by mental health literacy, self-stigma, and symptom misinterpretation^14,17^, as well as sociodemographic differences – most consistently, the higher likelihood of women using mental health services compared to men^18–20^. These dynamics reflect broader behavioral-health models that highlight the role of patient beliefs, perceived need, and illness representations in shaping treatment engagement.

In parallel, mental healthcare has increasingly emphasized patient-centered care, recognizing that patient preferences and experiences critically influence adherence and outcomes^21^. A meta-analytic review by McHugh and colleagues showed that most patients with depressive disorders prefer psychotherapy over antidepressant medication^22^, even though pharmacotherapy demonstrates a small but statistically significant efficacy advantage^23^. Patients often view psychotherapy as more likely to address the underlying causes of their depression^24^, and population-based data indicate high satisfaction among those receiving it^25^. Taken together, these findings suggest that **patients’ subjective evaluations of therapy**—not only clinical or system-level characteristics—are essential for understanding real-world treatment pathways.

Despite this, current evidence on treatment utilization in Germany is fragmented and often outdated. For instance, Freytag et al. (2015) analyzed health insurance data and reported that 51.6% of outpatients with depression received antidepressant pharmacotherapy, 7.8% received psychotherapy, and 13.8% received no treatment at all^26^. However, such administrative data do not capture patient experiences, unmet needs, or reasons for non-utilization. Self-reports, in contrast, can provide insights not reflected in billing or registry data. A study by Evans et al.^27^ demonstrated that patient self-reports of healthcare utilization show acceptable validity over the short and medium term. Given dynamic societal changes in mental health literacy, stigma, and help-seeking – exacerbated by global events such as the COVID-19 pandemic^28,29^ – relying solely on older administrative data risks misrepresenting current treatment patterns. In summary, while Germany offers a comprehensive range of guideline-based treatment options, significant knowledge gaps persist regarding how patients with depression actually engage with these services, how utilization varies across sociodemographic groups, and how patients themselves evaluate the helpfulness of different treatments. These gaps limit the ability to develop targeted strategies for reducing unmet need and improving access to effective care.

The present study therefore aims to: (i) describe the current use of psychotherapy, pharmacotherapy, and their combination among adults with diagnosed depression who report acute or residual symptoms; (ii) identify sociodemographic factors associated with the utilization of these guideline-based therapeutic options; and (iii) examine patients’ evaluations of their therapy to determine whether psychotherapy or pharmacotherapy is perceived as more helpful. To address these questions, we use a nationally representative sample of the German general population. Based on prior literature, we hypothesize that approximately half to two-thirds of the individuals with a diagnosed depression report receiving guideline-based treatment options, and that sociodemographic factors such as sex and age are associated with treatment utilization.

## Methods

### Design

Five cross-sectional, annual surveys were conducted between 2018 and 2022. The Ethics Committee of the Department of Medicine at Goethe University Frankfurt (Germany) confirmed that ethical review and approval were not required for this study in accordance with the local legislation and institutional requirements (reference number 2023 − 1174, 17 February 2023), and a written appraisal waiver was granted.

All study procedures were conducted in accordance with relevant guidelines, including the Declaration of Helsinki, and complied with ethical standards of the International Code of Marketing and Social Research Practice (International Chamber of Commerce, ICC) and the European Society for Opinion and Marketing Research (ESOMAR).

### Procedures

Data were collected as part of a population-representative survey of the German resident population aged 18–69 years. The surveys were conducted online by a commissioned, certified (ISO 26362) survey company (Respondi AG, Hürth, Germany). The sample was drawn using a multi-layered quota selection based on self-reported sex, age, and federal state (or groupings of federal states), in accordance with current population statistics from the Federal Statistical Office. Data collection was anonymous, and no personally identifying information was recorded or stored. All participants were registered in the Access Panel and provided written informed consent or assent to participate. Participants received an expense allowance in the form of points (equivalent to €1) redeemable for online purchases.

### Measures

Data were collected using a self-report questionnaire that included items on sociodemographic characteristics, knowledge about depression, personal experiences, assessments, and treatment preferences. All survey items were constructed ad hoc for the purposes of this study, using lay language to ensure comprehensibility in the general population. Participants were asked whether they had been affected by a depressive disorder themselves or knew someone affected (“Have you ever had contact with depression?; response options: “Yes I have been diagnosed with depression” / “Yes, I think I have had depression before, but without a diagnosis”/ „Yes, a relative or friend has been diagnosed with depression”/ “No, I have no direct connection to depression”). Participants also reported the current severity of their depressive symptoms (“How severely are you currently affected by depression?”; response options: “I am currently in a depressive episode”/ “I currently have some residual symptoms, but I am no longer in an acute depressive episode”/ “I currently have no depressive symptoms”). Respondents indicated whether they were using or had previously used various treatment options, including psychotherapy, antidepressant medication, online self-management programs, support groups, or alternative approaches such as meditation, hiking, or relaxation exercises (Response options: “Yes, I am currently using it”/ “Yes, I have used it before”/ “No, I have never used it”). From 2019 to 2022, participants who were undergoing treatment were additionally asked to rate how helpful they perceived their current therapy to be on a four-point scale (1 = not helpful at all, 2 = rather not helpful, 3 = somewhat helpful, 4 = helpful).

The present analyses focused on the self-reported use of psychotherapy, antidepressant medication, or their combination, which constitute the core components of guideline-based treatment for unipolar depression^1^.Individuals who reported receiving neither psychotherapy nor antidepressant medication were classified as having received “no guideline-based therapy”. Accordingly, no complex treatment pathways were evaluated.

### Study sample

Across the five survey waves, a total of 25.513 individuals participated. For the present analysis, we included respondents who self-reported having been professionally diagnosed with depression (*n* = 5.666; “Yes, I have been diagnosed with depression”) and who indicated either acute or residual symptoms (*n* = 3.416, “I am currently in a depressive episode” or “I currently have some residual symptoms but am no longer in an acute depressive episode”). These participants were asked about their current use of various treatment options for their condition. The sample was categorized into five age groups: 18–29, 30–39, 40–49, 50–59, and 60–69 years.

### Statistical analysis

Data analysis was performed using R studio^30^ (Version 2022.07.1 + 554) and SPSS (Version 28.0.1.0(142)). A significance level of α = 0.05 was applied for all analyses. To adjust the sample to the German population, data were weighted for age and sex according to the national administrative statistics. Weighting may result in slight deviations in totals, as figures are rounded to whole numbers.

We evaluated the use of various therapy options and the influence of sex and age on treatment utilization. Female participants and the 30–39 year age group were used as reference categories, allowing comparisons between middle-aged, younger (18–29 years), and older individuals (40–49, 50–59, 60–69 years). Prevalence rates were calculated using simple crosstabulations, and sociodemographic characteristics and therapy utilization are presented as weighted percentages. Differences in treatment use by sex and age were assessed with Pearson’s chi-square test. Logistic regression (odds ratios with 95% confidence intervals) was used to quantify associations between sociodemographic characteristics and therapy use.

Mean values were used to assess participants’ perceived helpfulness of current therapies. Participants receiving combination therapy (psychotherapy and antidepressants) evaluated each therapy separately. Differences in mean helpfulness ratings between psychotherapy and pharmacotherapy were tested using a two-tailed t-test.

## Results

### Sample characteristics

Of the total *n* = 25.513 respondents, *n* = 5.666 reported having ever been diagnosed with depression, corresponding to a lifetime prevalence of 22.2% in the present sample of the German general population. Among these individuals, *n* = 3.429 reported either acute (*n* = 1.084) or residual symptoms (*n* = 2.345) at the time of the surveys and provided information about their treatment for their current depressive episode. After weighting the data for age and sex according to national administrative statistics, the analytic sample comprised *n* = 3.416 participans, of whom *n* = 1.798 (52.6%) were female and *n* = 1.618 (47.4%) were male.

### Treatment utilization

Table 1 presents the distribution of current treatment utilization among individuals affected by depression, stratified by sex and age group. Overall, nearly two-thirds of participants (63.4%) reported using a guideline-based therapy: 13.5% received psychotherapy, 26.5% used antidepressant medication, and 23.4% reported a combination of both. Just over one-third (36.6%) indicated that they were not currently using psychotherapy or antidepressant medication for their depressive episode. Among these, only 1.8% of participants reported that they had never received psychotherapy or antidepressant medication in their lifetime.

**Table 1:** Utilization of treatment options for depression (self-reported, *n* = 3416 individuals with a diagnosed depression with acute or residual symptoms) by age and sex.

		*n*/*N* (%)	OR	[95% CI]		*p*	Test statistics
Guideline-based therapy		2166/3416 (63,4)					
Sex	f (= Ref.)	1150/1798 (64,0)	*1.00*	-	-	-	*X*^*2*^_*1*_ ***=*** *0.5*,* p = .502*
	m	1016/1618 (62,8)	0.94	0.82	1.08	0.402	
Age	18-29y	319/544 (58,6)	0.93	0.74	1.19	0.583	***X***^***2***^_***4***_ ***= 15.8***,*** p = .003***
	30–39 (= Ref.)	344/569 (60,5)	*1.00*	-	-	-	
	40–49	430/679 (63,3)	1.12	0.89	1.41	0.333	
	50–59	706/1043 (67,7)	**1.37**	**1.10**	**1.69**	**0.004**	
	60–69	367/582 (63,0)	1.12	0.88	1.42	0.355	
Psychotherapy		462/3416 (13,5)					
Sex	f (= Ref.)	247/1798 (13,7)	*1.00*	-	-	-	*X*^*2*^_*1*_ *= 0.1*,* p = .739*
	m	215/1618 (13,3)	0.96	0.78	1.17	0.675	
Age	18-29y	116/544 (21,3)	1.22	0.91	1.64	0.194	***X***^***2***^_***4*** _ = **64.6**, *** p < .001***
	30–39 (= Ref.)	103/569 (18,1)	*1.00*	-	-	-	
	40–49	87/679 (12,8)	**0.66**	**0.48**	**0.90**	**0.009**	
	50–59	112/1043 (10,7)	**0.55**	**0.41**	**0.73**	**< 0.001**	
	60–69	43/582 (7,4)	**0.36**	**0.24**	**0.52**	**< 0.001**	
Antidepressants		904/3416 (26,5)					
Sex	f (= Ref.)	495/1798 (27,5)	*1.00*	-	-	-	*X*^*2*^_*1*_ *= 2.1*,* p = .149*
	m	409/1618 (25,3)	0.90	0.77	1.06	0.201	
Age	18-29y	81/544 (14,9)	0.74	0.54	1.01	0.056	***X***^***2***^_***4***_ ***= 92.6***,*** p < .001***
	30–39 (= Ref.)	110/569 (19,3)	*1.00*	-	-	-	
	40–49	185/679 (27,3)	**1.56**	**1.19**	**2.04**	**0.001**	
	50–59	312/1043 (29,9)	**1.78**	**1.39**	**2.28**	**< 0.001**	
	60–69	216/582 (37,1)	**2.46**	**1.89**	**3.23**	**< 0.001**	
Psychotherapy + antidepressants		800/3416 (23,4)					
Sex	f (= Ref.)	408/1798 (22,7)	*1.00*	-	-	-	*X*^*2*^_*1*_ *= 1.0*,* p = .309*
	m	392/1618 (24,2)	1.06	0.90	1.24	0.482	
Age	18-29y	122/543 (22,4)	0.97	0.74	1.29	0.858	***X***^***2***^_***4*** _***= 16.2,*** *** p = .003***
	30–39 (= Ref.)	131/569 (23,0)	*1.00*	-	-	-	
	40–49	158/679 (23,3)	1.01	0.77	1.32	0.944	
	50–59	282/1043 (27,0)	1.24	0.97	1.57	0.083	
	60–69	107/582 (18,4)	0.77	0.58	1.02	0.072	
No psychotherapy or antidepressants		1250/3416 (36,6)					
Sex	f (= Ref.)	648/1798 (36,0)	*1.00*	-	-	-	*X*^*2*^_*1*_ = *0.5*,* p = .502*
	m	602/1618 (37,2)	1.06	0.92	1.22	0.402	
Age	18-29y	225/544 (41,4)	1.07	0.84	1.36	0.583	***X***^***2***^_***4***_***= 15.8***, *** p = .003***
	30–39 (= Ref.)	225/569 (39,5)	*1.00*	-	-	-	
	40–49	249/679 (36,7)	0.89	0.71	1.12	0.333	
	50–59	337/1043 (32,3)	**0.73**	**0.59**	**0.91**	**0.004**	
	60–69	216/583 (37,0)	0.89	0.70	1.13	0.355	

*Notes.* Results of multiple logistic regression analyses and chi-square analyses of the use of therapy options from 2018 to 2022, percentages weighted by age, sex, and state.

f = female, m = male, age in years, ref.= reference category of logistic regression, OR = odds ratio, CI = confidence interval, X^2^ = Chi-square.

Statistically significant results (*p* < .05) are **bold**.

### Differences based on sociodemographic differences

The overall proportions of participants using guideline-based therapy were similar between the sexes, with 64.0% of females and 62.8% of males reporting such treatment. Differences between the sexes within the subgroups of the guideline-based therapy were small and not statistically significant: 13.7% of females versus 13.3% of males used psychotherapy, 27.5% versus 25.3% used antidepressants, and 22.7% versus 24.2% used a combination of both. Likewise, there was no significant difference in the proportion of females (36.0%) and males (37.2%) using non-guideline-based therapy.

Regarding age, the overall likelihood of receiving guideline-based therapy for the current depressive episode generally increased with age, from 58.6% in the youngest group (18–29 years) to 67.7% in the 50–59 year group, before declining slightly to 63.0% in the 60-69-year group. Subgroup analysis revealed that psychotherapy utilization decreased significantly with increasing age [*18–29 years: OR = 1.22*,* 95% CI 0.91–1.64; 60–69 years: OR = 0.36*,* 95% CI 0.24–0.52*; *p* < .001], with prevalence dropping from 21.3% in the youngest group to 7.4% in the oldest group. In contrast, antidepressant use increased significantly with age [*18–29 years: 14.9%*,* OR = 0.74*,* 95% CI 0.54–1.01; 60–69 years: 37.1%*,* OR = 2.46*,* 95% CI 1.89–3.23*; *p* < .001]. Combination therapy also showed a significant increase with age *(X² = 16.2*,* p = .003)*, although there were no significant subgroup differences in relation to the reference group (30–39 years).

Among the participants not using psychotherapy nor pharmacotherapy, the highest proportion was observed in the youngest age group (18–29 years), with just over two in five individuals (41.4%) not using one of the pillars of guideline-based treatment for depression.

### Evaluation and comparison of therapy options

Participants rated the helpfulness of their current treatment on a scale from 1 (not helpful at all) to 4 (helpful); all mean values are shown in Table 2. Those receiving psychotherapy rated their treatment as helpful (mean ${ {\bar{{\rm X}}}}$ = 3.6), while participants on antidepressant medication rated it as somewhat helpful to helpful (mean ${ {\bar{{\rm X}}}}$ = 3.5). There was no statistically significant difference between satisfaction with psychotherapy versus antidepressants when used as monotherapy (t(1.080) = 1.29, *p* = .199, 95% CI = [ -0.03, 0.13]).

**Table 2:** Evaluation of the currently used therapy option.

	*N*	Mean ${ {\bar{{\rm X}}}}$
Monotherapy with psychotherapy	367/1723	3,6
Monotherapy with antidepressants	715/1723	3,5
Combination therapy		
Psychotherapy	641/1723	3,6
Antidepressants	641/1723	3,4

*Notes* results of therapy evaluation (from 2019 to 2022) by means of a Likert-Skala from one to four (1 = not helpful at all, 2 = rather not helpful, 3 = somewhat helpful, 4 = helpful).

The participants rated the therapy options of the combination therapy separately.

Participants receiving combination therapy rated each therapy individually: psychotherapy (${ {\bar{{\rm X}}}}$ = 3.6) and antidepressants (${ {\bar{{\rm X}}}}$ = 3.4). No statistically significant difference in perceived helpfulness was observed between the two therapy modalities when in combination.

## Discussion

This study provides novel, population-representative evidence on the utilization of depression therapies among adults with a self-reported diagnosis of depression in Germany. Overall, 63.4% of individuals with current depressive episodes or residual symptoms reported receiving guideline-based therapy. Age emerged as a key determinant of treatment use, with the likelihood of engaging in guideline-based treatments increasing with age. Notably, the youngest age group (18–29 years) had the highest proportion of individuals not receiving either psychotherapy or pharmacotherapy, underscoring a critical gap in the utilization of guideline-based treatment and pointing to a critical public health concern for younger adults. Contrary to our expectations, sex was not associated with treatment utilization, and participants’ ratings of the helpfulness of psychotherapy versus antidepressants did not differ, suggesting comparable perceived benefits across treatment modalities.

The results of this study align with international findings indicating that approximately 50–70% of individuals diagnosed with depression in high-income countries receive minimally appropriate, guideline-concordant treatment^19,20,31^. In Germany, a previous nationwide monitoring study by Freytag et al. (2015) reported higher rates of antidepressant monotherapy among outpatients (46.2%), but lower utilization of psychotherapy (7.3%) and combination therapy (5.4%) compared with our findings. That study also documented a smaller proportion of individuals receiving no guideline-based treatment (13.6%), defined as neither adequate psychotherapy nor adequate antidepressant pharmacotherapy^26^. Several factors may account for these discrepancies. Freytag et al. relied on AOK health insurance billing data, capturing physician-prescribed treatments primarily related to the initial manifestation of depression. Individuals who did not receive reimbursed therapy were therefore underrepresented. In contrast, our population-based survey included all adults with a self-reported depression diagnosis – whether initial or recurrent – and fully captured those not receiving psychotherapy or pharmacotherapy in the “no guideline-based therapy” category. Various factors may explain the non-utilization of guideline-based therapy (e.g., delayed treatment initiation, nonresponse, or refusal), but these cannot be determined from our data.

Depression is known to occur about twice as often in women as in men^32–34^, but this pattern was less pronounced in our sample because only individuals with a self-reported diagnosis of depression were included. Although previous research has shown that women are more likely than men to seek and use treatment for depression^20,31,35,36^, we did not observe such differences. Our findings indicate that, in this sample, sex did not appear to influence the choice of treatment for depression.

This study highlights pronounced age-related disparities in the use of guideline-based therapies for depression, with important public health implications. Individuals aged 50–59 years were most likely to receive psychotherapy, pharmacotherapy, or a combination for current depressive episodes or residual symptoms, consistent with international evidence of higher proportions of receiving adequate treatment in middle- to older-aged adults^19,20^. In contrast, younger adults (18–29 years) had the highest proportion not receiving antidepressant pharmacotherapy, revealing a critical gap in the utilization of guideline-based care despite greater public awareness and acceptance of mental health treatment^37^. Limited autonomy and dependence on family or significant others may partly explain difficulties regarding initiating and continuing treatment in this age group^38^. Notably, older adults (50–69 years) were less likely to use psychotherapy, reflecting persistent attitudinal and systemic barriers. Some perceive depression as a normal part of aging^39^ or associate seeking help with personal weakness, and older individuals may also struggle with the age gap when therapists are younger^40^. On the supply side, primary care physicians may misattribute depressive symptoms to aging or assume that older patients are untreatable^40,41^. These combined demand- and supply-side factors indicate that both younger and older adults remain underserved, albeit for different reasons. Addressing these gaps is crucial, as insufficient engagement in guideline-based treatments can lead to persistent depressive symptoms and the development of treatment-resistant depression, which affects approximately one-third of patients and imposes substantial societal and clinical burdens [9, Fiorillo et al., 2025]. Depression and other mental disorders during adolescence and young adulthood also increase the risk of later psychiatric problems, substance use, suicide attempts, and premature school dropout^42,43^. Targeted strategies are therefore urgently needed to improve access, engagement, and adherence to evidence-based depression treatments across age groups.

Previous research has shown that individuals generally prefer psychotherapy over antidepressant medication for treating depression^44^, often viewing psychotherapy as addressing the root cause while seeing antidepressants as non-causal^21^. Accordingly, we expected psychotherapy to be rated as more helpful. However, participants in our study reported similar satisfaction with both psychotherapy and antidepressant treatment. This finding suggests that misconceptions about antidepressants persist^45^, which may pose a major barrier to optimizing depression care and ensuring patients access effective treatment^46^.

The main strength of this study is its five-year data collection, which enhances the representativeness of the findings. In addition, the sample is representative of the German general population. Nevertheless, several limitations should be considered when interpreting the results. First, data were collected via an online self-report survey, which may introduce selection bias. Individuals with low digital literacy or severe depressive symptoms may have been less likely to participate, potentially leading to underrepresentation of these groups and an underestimation of treatment utilization, as more severe depression is associated with higher treatment rates (Freytag et al.^26^). Consequently, the findings may further not be fully generalizable to these groups. Self-report data may also be affected by recall or self-assessment bias. However, online surveys ensure anonymity, which can facilitate honest reporting, and self-reported treatment utilization has shown good short- to medium-term validity^27,47,48^. Second, depression diagnosis, as well as current and residual symptoms, was assessed using lay-language self-report questions. We were therefore unable to distinguish between treatment-resistant and difficult-to-treat depression or exclude individuals with depressive symptoms stemming from other psychiatric conditions (e.g., bipolar or schizoaffective disorders). Although these conditions are less common in the general population, this potential heterogeneity warrants caution. Third, the study relied on ad hoc self-report questions rather than validated clinical scales to assess depressive symptoms and treatment status. While this approach enabled efficient assessment in a large population sample, it may limit comparability with studies using standardized diagnostic instruments and could introduce measurement bias. Fourth, we could not assess adequacy of care because therapy quality, duration, or adherence were not captured, and our operational definition of guideline-based therapy may oversimplify complex treatment pathways. Moreover, we did not assess complementary evidence-based treatments recommended in clinical guidelines (e.g., exercise, light therapy, electroconvulsive therapy), making it impossible to determine their utilization. Our analysis focused solely on current treatment use; thus, we cannot infer which treatments participants may have received in the past. Severity of depression – an important determinant of treatment choice – was also not measured. Fifth, respondents using both psychotherapy and antidepressant medication evaluated each treatment separately, preventing direct analysis of combination therapy as a distinct guideline-based modality. As a result, no conclusions can be drawn regarding perceived helpfulness of combination treatment.

This study aimed to improve the understanding of guideline-based treatment utilization among individuals with depressive disorders in Germany. Despite the availability of effective treatments such as psychotherapy and antidepressant medication, a substantial proportion of individuals with current depressive symptoms were not using either option. While treatment patterns did not differ by sex, age emerged as a key sociodemographic factor influencing both the likelihood of treatment use and the type of therapy selected. Our findings point towards a pronounced treatment gap among younger adults. Inadequate treatment increases the risk of persistent depressive symptoms and the development of treatment-resistant depression^9^. Because age strongly influences treatment utilization, interventions may need to be tailored to different life stages. Strengthening mental health literacy in youth is a key lever for improving both individual and public health outcomes^49^. Evidence suggests that school- and community-based programs can enhance mental health literacy among adolescents^50^, although findings on their long-term effectiveness remain mixed^51^. For young adults, additional promising approaches include digital therapy consultations, mental health apps, and greater visibility of prevention, destigmatization, and psychoeducation through social media. Emerging evidence further indicates that AI-driven conversational agents may help reduce depressive symptoms in this age group^52^. For older adults, interventions that enhance the mental health literacy of their relatives may improve support for seeking care^53^. Such efforts should prioritize expanding access to psychotherapy in addition to pharmacotherapy. Further research is needed to better understand and reduce these age-related disparities in depression care. Given the rising societal burden of depression, there is an urgent need for chronic care models and individualized treatment pathways, complemented by targeted strategies for treatment-resistant depression^54^. Similar to other chronic conditions, chronic care models provide structured, coordinated care and help enhance patients’ health. For depression, such programs should not only address acute episodes but also support ongoing self-management of residual symptoms and ensure rapid escalation of treatment when necessary.

## Data Availability

The datasets analysed during the current study are available from the corresponding author on reasonable request.
